# Dynamic back analysis of soil deformation during the construction of deep cantilever foundation pits

**DOI:** 10.1038/s41598-022-17513-4

**Published:** 2022-07-30

**Authors:** Jian Zhang, Guangxuan Qiao, Tugen Feng, Yihe Zhao, Chunbin Zhang

**Affiliations:** 1 Key Laboratory of Ministry of Education for Geomechanics and Embankment Engineering, Hohai University, No. 1 Xikang Road, Nanjing, 210024 China; 2 China Railway (Shanghai) Investment Group Co. LTD, Shanghai, 200135 China

**Keywords:** Civil engineering, Computational science

## Abstract

Field monitoring of foundation pits alone cannot predict the future deformation of retaining structures. Numerical simulations can predict the deformation of foundation pits and the working state of retaining structures to avoid the risk of foundation pit damage in advance. Accurate inversion of the soil parameters used for simulation and prediction is a key step. The associated multivariable problem is transformed into a single-variable problem by using the interval influence coefficient. Soil layer weightings and excavation step weightings are introduced and exploited to optimize the calculation process, and the soil parameters are calculated through inversion based on the least squares method. Based on actual engineering, the excavation sequence is regarded as a progressive sequence for back analysis, and the parameters of each soil layer are calculated through dynamic calculations with the excavation process in a cycle comprising inversion, prediction, reinversion and reprediction. The soil parameters after inversion are used to predict the maximum value and the depth of the deep horizontal displacement of the retaining structure, which verified the feasibility of the back-analysis method. Compared with the results before inversion, after the final inversion, t the overall error of section 2 is reduced by 67.24%, the overall error of section 3 is reduced by 40.5%, and the overall error of section 4 is reduced by 35%. The prediction curves are all close to the monitoring displacement curves, which plays a good guiding role and ensures the safe construction of the foundation pit. A new effective idea is proposed for the inverse analysis of the composite formation parameters of the deep foundation pit engineering.

## Introduction

A cantilever retaining structure cannot penetrate an aquifer. During the excavation of a foundation pit, the groundwater level outside the pit drops due to dewatering in the pit; this phenomenon leads to nonuniform settlement of the surrounding surface, a surge at the bottom of the foundation pit, and cracks or other damage within the surrounding buildings. To ensure the safety of a foundation pit and surrounding structures, the deformation state of the retaining structure in the foundation pit is usually monitored. However, foundation pit monitoring can provide feedback on the deformation of the retaining structure only after each construction step; that is, monitoring alone cannot predict the future deformation of the structure. Hence, it is difficult to predict the types and components of possible risks in advance and to propose reasonable measures to ensure safe construction. The numerical simulation method can be used to study the deformation of a retaining structure throughout the foundation pit excavation process through simulations and calculations, and is thus an effective way to evaluate the reliability of the retaining structure^1–4^. Generally, when a numerical simulation is used to study the deformation of a foundation pit, the first step is to build an excavation model of the foundation pit with numerical software. Then, soil parameters obtained from laboratory tests or in situ field soil tests are assigned to the model, and finally, an excavation simulation is carried out to analyze the stress and deformation laws of soil around the foundation pit. However, the soil parameters measured in the laboratory or in the field and the actual soil parameters are often different^5,6^, resulting in large errors between the results of numerical simulations and the actual situation. Although the treatment of the bracings, modeling of the soil-structure interface and the constitutive law of the soil can also cause differences between the results of the numerical analysis and the actual situation (monitored results), for actual projects that consider an established numerical simulation model, the acquisition of reasonable soil parameters is crucial for accurately predicting deformation and ensuring safe construction^7–9^.

Based on actual monitoring data, the inversion analysis method can obtain the actual undisturbed stress or soil parameters of a project^10,11^ through many recursive cycles. During the inversion, the error between the positive calculated analysis value and the monitored value is minimized, and reasonable model parameters for the numerical simulation method are provided. This approach can be used to predict the deformation and stress of each part of a structure during the construction process^12^. Based on the analysis results, possible risks can be determined in advance and thus avoided within a reasonable range. Scholars worldwide have carried out several studies on the basis of back analysis of geotechnical engineering. Zhu et al.^13^ first proposed the application of dynamic inversion to a construction project and considered the influences of various working conditions on foundation pit deformation during excavation. Knaba et al.^14^ proposed a back analysis algorithm to determine the constitutive parameters of a geotechnical boundary value problem. However, this back analysis algorithm easily converges at a local optimum during solution. To improve the inversion prediction accuracy, Finno and Calvello^15^ optimized the U-CODE algorithm based on the Newton method and improved the prediction performance of a foundation pit supporting structure model. Tang and Kung^16^ introduced nonlinear optimization technology into a finite element program for numerical simulation. Ye et al.^17^ analyzed monitoring data to simulate the excavation of a foundation pit. Burridge et al.^18^ designed a linear stability inversion program. The above four inversion methods have achieved reasonable simulation results. With the emergence of big data and artificial intelligence, the use of intelligent algorithms to deduce soil parameters has gradually become more extensive. Based on a genetic algorithm combined with the least squares method, Zhu and Liu^19^ designed an intelligent inversion analysis method with strong global convergence. Gu and Gu^20^ designed an optimization back analysis calculation method to improve the objective function. Hashash et al.^21^ considered the parameter optimization method of adopting a genetic algorithm and self-learning simulation back analysis technology. Ran et al.^22^ adopted a back propagation neural network algorithm. The above three inversion methods not only ensure the inversion accuracy, but also improve the inversion efficiency. However, the inversion methods mentioned above cannot fully consider the influences of multiple soil layers and the excavation step on the horizontal displacement of the soil. Hence, for complex problems involving multiple parameters and multiple excavation steps, the optimization efficiency is low, and the calculations are complex. It is therefore difficult to achieve global optimization due to the interaction among multiple factors.

Based on actual monitoring data of the foundation pit of the Baguazhou open excavation section of the river crossing the passage of Heyan Road in Nanjing, this paper presents a cyclic calculation method for dynamic optimization inversion parameters and applies the optimized parameters to a numerical simulation to accurately predict the deep horizontal displacement of the soil; the results provide engineering guidance for the safe construction of a cantilever retaining structure. According to the field conditions and parameter sensitivity analysis, the maximum value and the location of the deep horizontal displacement of the soil layer is determined as the inversion target, and the ratio of the elastic modulus *E* to the compressibility modulus *E*_*s*_, called the elasticity-compressibility ratio *ξ*, is determined as the inversion parameter. Four sections are selected as typical sections. According to the excavation depth from shallow to deep, four analyses, namely, inversion, prediction, reinversion and reprediction, are carried out in succession. In these analyses, the inversion parameters of the soil layers in the former section are taken as the prediction parameters of the next section to study the deformation of the retaining structure and guide the engineering construction. If the number of soil layers in the next section increases, the parameters of the new soil layer are calculated by another inversion based on the monitoring data, and the deformation of the retaining structure in the next section is predicted. By calculating the interval influence coefficient of each soil layer parameter on the deep horizontal displacement of the soil, the influences of multiple variables on the objective function are expressed by weighting^23^, and interval optimization is carried out to determine the optimal soil parameters.

## Back analysis calculation

**Figure 1:**
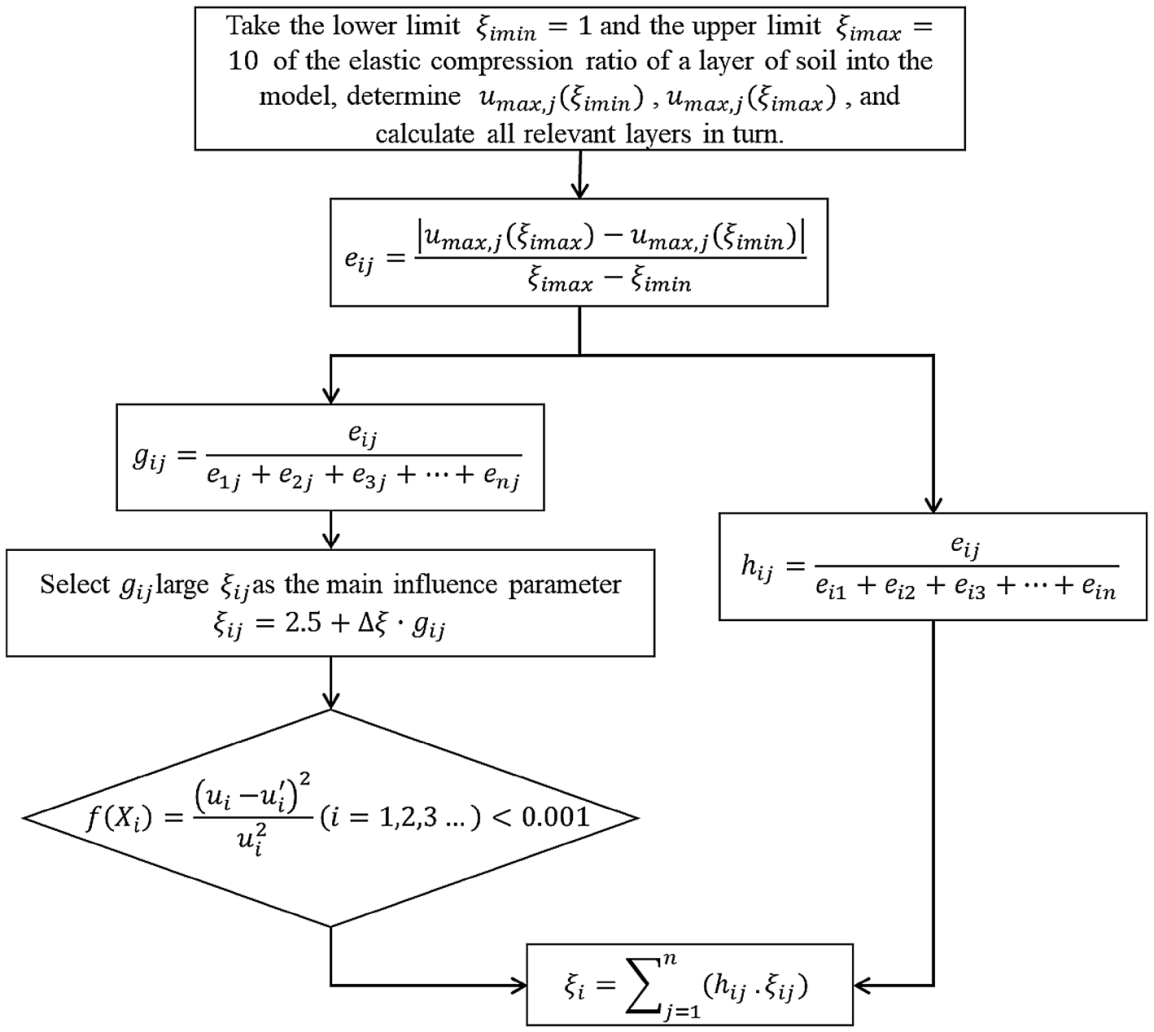
Inversion calculation flow chart.

The back analysis and calculation processes are as follows: first, establish a numerical model of the foundation pit being excavated; second, determine the inversion target and inversion parameters; third, define the interval influence coefficient of each soil layer according to the degrees of influence of the inversion parameters of each soil layer on the inversion target; and fourth, determine the final inversion parameters based on the inversion evaluation index and take them as the prediction parameters for the next section of construction until the parameter inversion and prediction of each soil layer in all sections are completed. Figure 1 shows he overall flowchart, and the specific steps are as follows:

1. Basic assumptions: The initial value of the inversion parameter *ξ*_*i*_ of each soil layer is a constant *k*; the inversion target is the parameter *u*; the soil layer has *n* layers, and the excavation step has *m* steps.
2. Model establishment: The section with the greatest thickness of the soft soil layer or the greatest excavation width in the construction section is selected as the typical section. ABAQUS is used to establish numerical simulation analysis models of the typical sections of the foundation pit.
3. Initial calculation: Take the initial inversion parameters of each soil layer as the constant *k*, and carry out the initial finite element calculation of the whole construction process.
4. Determining the maximum value *u*_*max,j*_ of the inversion target: Assume that the upper and lower limits of the inversion parameters of the different soil layers *i*(*i* = 1, 2, 3,…, *n*) on a typical section are *ξ*_*imax*_ and *ξ*_*imin*_, respectively, which can be used to calculate the maximum value of *u* under different excavation steps *j* (*j* = 1, 2, 3,…, *m*), recorded as *u*_*max,j*_ (*ξ*_*imax*_) and *u*_*max,j*_ (*ξ*_*imin*_).
5. Determining the influence coefficient: Define the interval influence coefficient *e*_*ij*_ of the maximum value of the inversion target in excavation step *j* of soil layer *i*.
  1 $$ e_{ij} = \frac{{\left| {u_{\max ,j} \left( {\xi_{i\max } } \right) - u_{\max ,j} \left( {\xi_{i\min } } \right)} \right|}}{{\xi_{i\max } - \xi_{i\min } }} $$
  The coefficient can directly reflect the influence of the inversion parameters of soil layer *i* on the inversion target in excavation step *j*.
6. Soil layer weighting: For excavation step *j*, the weighting *g*_*ij*_ of different soil layers can be expressed as:
  2 $$ g_{ij} = \frac{{e_{ij} }}{{e_{1j} + e_{2j} + \cdot \cdot \cdot + e_{nj} }} $$
  where *g*_*ij*_ reflects the influence degree of soil layer *i* on the inversion target in excavation step *j*. The greater the weighting *g*_*ij*_ is, the greater the influence degree. The inversion parameter *ξ*_*ij*_ (the inversion parameter *ξ* of step *j* and soil layer *i*) of the soil layer corresponding to the maximum weighting *g*_*ij*_ is selected as the main influencing parameter. The bisection method is used to reduce the range of *ξ*_*ij*_, and the parameters of the other soil layers are synchronously reduced according to the soil layer weighting *g*_*ij*_. In the process of adjustment, the determined parameters are input into the finite element model for numerical calculation.
7. Evaluation index assessment: According to the least squares method, the evaluation function is defined as shown in Eq. (3). When *f*(*ξ*_*ij*_) < = 0.001, the evaluation index is met, and the calculation is ended; when *f*(*ξ*_*ij*_) > 0.001, the bisection method is used to narrow the range of *ξ*_*ij*_ until the evaluation index is met.
  3 $$ f\left( {\xi_{ij} } \right) = \frac{{\left( {u_{i} - u_{i}^{^{\prime}} } \right)^{2} }}{{u_{i}^{2} }}\quad \left( {i = 1,2,3 \ldots ,n} \right) $$
  where *u*_*i*_ is the inversion target, $u_{i}^{^{\prime}}$ represents the field monitoring data, and *i* is a unique soil layer.
8. Excavation step weighting: For soil layer *i*, the weighting *h*_*ij*_ of different excavation steps can be expressed as:
  4 $$ h_{ij} = \frac{{e_{ij} }}{{e_{i1} + e_{i2} + \cdot \cdot \cdot + e_{im} }} $$
  The value of *ξ*_*i*_ for each excavation step in soil layer *i* is merged according to *h*_*ij*_.
  5 $$ \xi_{i} = \sum\limits_{j = 1}^{m} {h_{ij} \cdot \xi_{ij} } $$
9. Guide the construction: Based on the inversion results of the soil parameters, the numerical model is used to predict the deformation of the retaining structure of the foundation pit being excavated and to guide the excavation and construction of the foundation pit.

If the next section involves an additional soil layer, the new soil layer of the next section is predicted based on the results of the soil layer inversion parameters in the current section. Steps (3)–(9) are repeated, and the cyclic calculation, namely, the inversion, prediction, reinversion and reprediction, are carried out in combination with the monitoring data until the parameter inversion and prediction of each soil layer in all sections are completed.

## Project overview and calculation model

### Project overview

**Figure 2:**
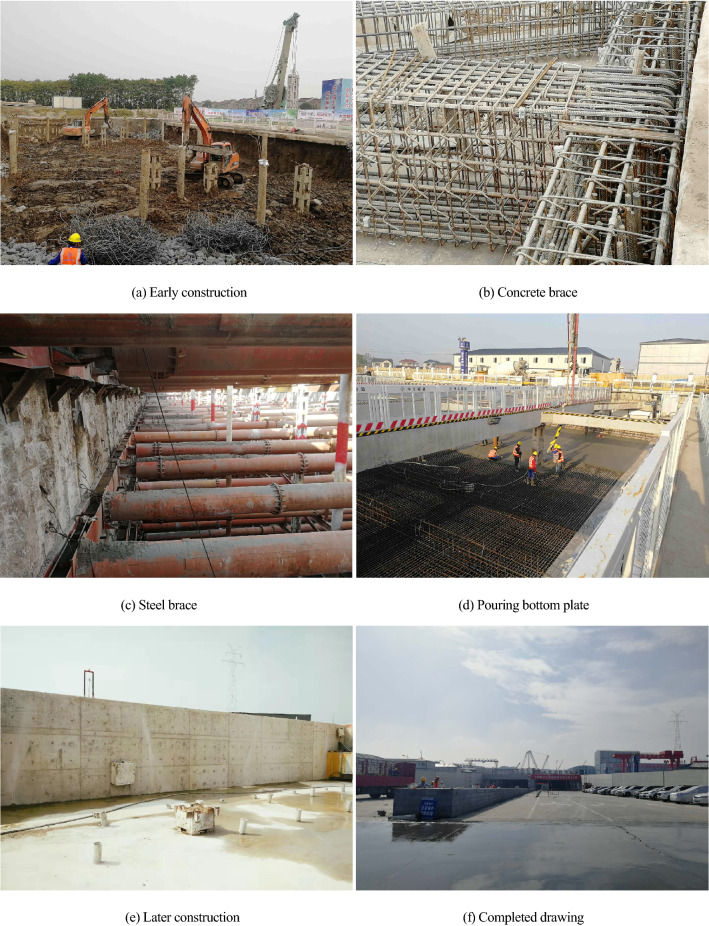
Construction process of foundation pit. (**a**) Early construction. (**b**) Concrete brace. (**c**) Steel brace. (**d**) Pouring bottom plate. (**e**) Later construction. (**f**) Completed drawing.

The river crossing the passage of Heyan Road, which stretches from the intersection of Heyan Road and Yanheng Road and to the intersection of Baguazhou and Puyi Road, is located in Qixia district, Nanjing. The total length of the Baguazhou open excavation section is 440 m, the width of the foundation pit is 33–54 m, and the maximum depth of the foundation pit is 25.7 m. According to the excavation depth, a variety of retaining structure types are selected, mainly including soil–cement mixed walls (SMWs, with a cross-sectional diameter of 650 mm) and diaphragm walls (with thicknesses of 600 mm and 800 mm). The bracing structure adopts both a concrete brace and steel brace. In this foundation pit project, a cantilever retaining structure is used, and thus, the hydraulic power connection between the inside and outside of the foundation pit is not completely cut off. Hence, the retaining structure cannot provide complete waterproofing, and the deformation of the foundation pit is greatly affected by dewatering. The site construction process is shown in Fig. 2.

**Figure 3:**
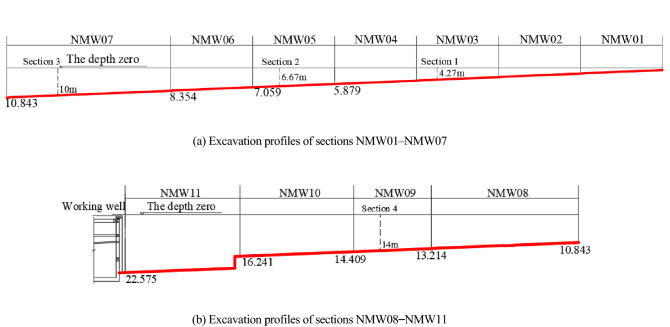
Excavation profiles of sections NMW01–NMW11. (**a**) Excavation profiles of sections NMW01–NMW07. (**b**) Excavation profiles of sections NMW08–NMW11.

The cut and cover method is adopted to excavate the foundation pit. The foundation pit is divided into 11 construction sections along the longitudinal direction, and the excavation sequences are numbered from NMW01 to NMW11 (the excavation depth rises from shallow to deep, and the number of soil layers within the excavation range increases gradually). The foundation pit is excavated layer by layer from the top to the bottom, and the cross brace is installed at the time of excavation. The excavation result for each construction section is shown in Fig. 3, where the red line in the figures is the final excavated surface and the numbers describe the depth of the foundation pit.

The project is located in the flood plain area of the Yangtze River. The foundation soil is composed of Quaternary (Holocene and upper Pleistocene) sediment. According to the ages, causes of formation and physical and mechanical indexes of the soil layers, combined with the project excavation details, the soil is divided into six main layers, of which the fifth layer is divided into two sublayers; the specific parameters obtained from the geological exploration report are shown in Table 1.

**Table 1:** Soil layer parameters.

Name	Density (g/cm^3^)	*E*_*s*_ (MPa)	*υ*	*c* (kPa)	*φ* (°)
Clay	1.82	3.95	0.33	16.1	15.25
Muddy silty clay	1.77	3.11	0.36	9.42	12.04
Silt with sand	1.88	7.42	0.28	6.65	21.83
Sand with silt	1.94	11.51	0.26	2.64	30.5
Silty sand	1.92	13.8	0.28	2.24	33.66
Silty sand	1.96	13.72	0.28	2.39	33.24

### Computational model

**Figure 4:**
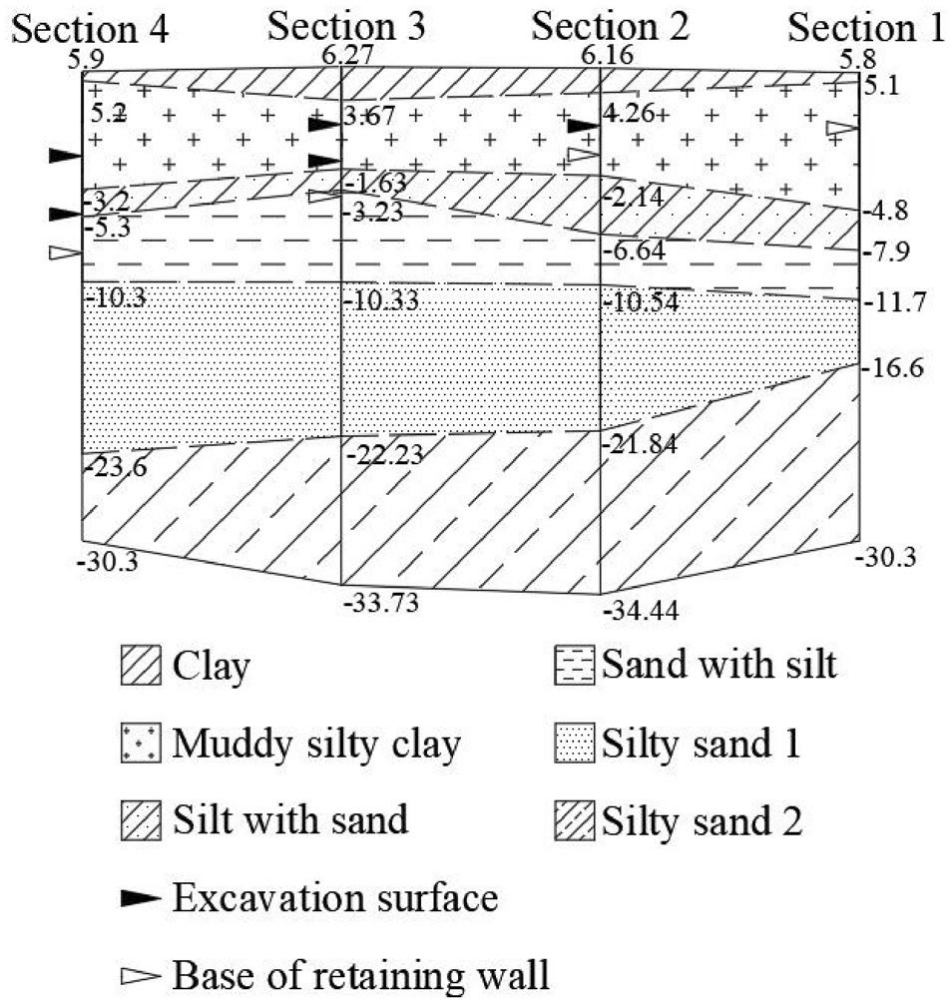
Excavation steps and soil layers.

When the ratio of the length to the width of the foundation pit is greater than 4, the distributions and values of the displacement, settlement and internal force calculated by the 3D model are essentially consistent with those calculated by the 2D plane model^24^. The open excavation section of the Heyan Road foundation pit is far longer than the width, so this configuration can be simplified as a 2D problem for the simulation. Four sections with the greatest thickness of the soft soil layer or the greatest excavation width are selected as the control sections for the 11 excavation sections. The excavation depths of the sections and the number of parameters of the soil layer successively increase. A 2D finite element model of the four sections is established. The excavation steps and soil layers of each section are shown in Fig. 4. According to the excavation depth, the bracing and retaining structures employed in each section are shown in Table 2.

**Table 2:** Bracing and retaining methods.

Section number	Excavation steps	Depth (m)	Retaining structure	Bracing structure
1	1	4.27	Φ650 mm SMW, 10 m long pile	One concrete brace
2	2	4.46	Φ650 mm SMW, 14 m long pile	One concrete brace + one Φ609 mm steel brace
		6.67		
3	3	4.46	600 mm thick diaphragm wall + jet grouting piled water-stop curtain, 21 m deep wall	One concrete brace + two Φ609 mm steel braces
		7.26		
		10		
4	3	6.5	800 mm thick diaphragm wall + jet grouting piled water-stop curtain, 28 m deep wall	One concrete brace + two Φ800 mm steel braces
		11		
		14		

Due to the complexity of foundation pit engineering and the simplicity of the soil layer parameters provided in the geological exploration report, a numerical model^25^ that combines the elastic model and Mohr Coulomb model is selected, as this model can suitably consider the plasticity of the soil without making the parameters of the model too complex.

The influence range of the model boundary on the static response of the structure is approximately 3–5 times the plane size of the structure^26^, and the influence of the horizontal boundary of the model on the structure is far greater than the influence of the bottom boundary. Hence, for the calculation area of the model, the length of the pit in the transverse direction is approximately 5 times the excavation width of the foundation pit, and the breadth of the pit in the longitudinal direction is 3–5 times the excavation depth of the foundation pit. The detailed dimensions of the sections are shown in Table 3.

**Table 3:** Detailed dimensions of each section.

Section number	Excavation section	Excavation depth (m)	Excavation width (m)	Calculation area (m^2^)
1	NMW03	4.27	46.7	210 × 20
2	NMW05	6.67	48	240 × 35
3	NMW07	10	35	175 × 45
4	NMW09	14	34	170 × 50

Horizontal and normal constraints are applied to the bottom boundary of the calculation model; horizontal constraints are applied to both sides of the model; and the upper boundary is a free boundary without constraints. According to the geological exploration report, the groundwater level was 0.42–2.20 m deep throughout the project, with an elevation of 4.64–5.58 m, so the initial groundwater level is set to 0.42 m deep. Based on the actual dewatering scheme, the groundwater level is lowered in the pit to 1 m below the excavation surface before each excavation step.

**Figure 5:**
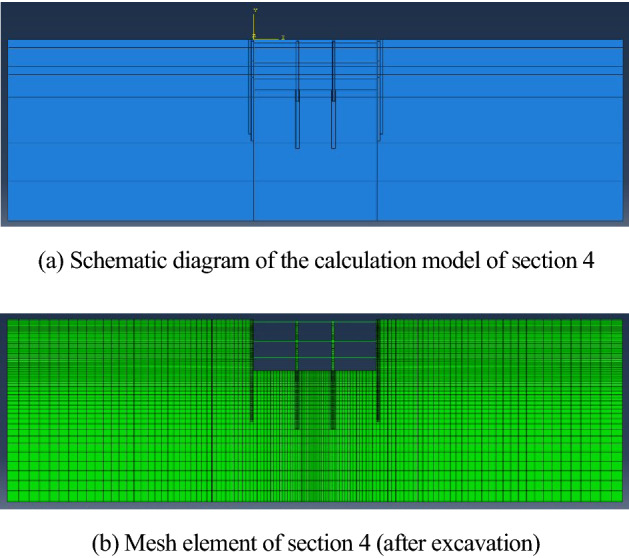
Calculation model and mesh element of section 4. (**a**) Schematic diagram of the calculation model of section 4. (**b**) Mesh element of section 4 (after excavation).

A solid element is used in this simulation, and leakage must be considered in the soil, so CPE4P is selected as the mesh element type. CPE4 is utilized as the mesh element of the retaining structure and pile, and the mesh element type employed for the bracing structure is the B21 linear beam element. Horizontal and normal constraints are applied to the bottom surface of soil boundary, *U*_*x*_ = *U*_*y*_ = 0; horizontal restraint is applied to both sides, *U*_*x*_ = 0. The calculation model and grid division of section 4 are shown in Fig. 5.

The main process of simulating the foundation pit excavation is as follows:

1. Balance the initial ground stress, and simulate the stable state of the soil before excavation;
2. Activate both the load and the retaining structure (Table 4);
3. Lower the groundwater level to 1 m below the excavation surface;
4. Excavate the soil in the foundation pit according to the excavation step;
5. Simulate the brace erections according to the construction scheme, and apply the preaxial force at the same time;
6. Repeat steps 3–5 until the excavation reaches the bottom of the foundation pit.

**Table 4:** Physical and mechanical parameters of the model.

Material	Density (g/cm^3^)	Modulus of deformation (MPa)	*υ*	*c* (kPa)	*φ* (°)
Diaphragm wall	2.5	30,000	0.2	–	–
Concrete brace	2.5	32,500	0.2	–	–
Steel brace	7.85	210,000	0.25	–	–
Jet grouting piled water-stop curtain	1.9	300	0.2	15	50
SMW	2.5	20,000	0.2	–	–

## Back analysis target and parameter

### Back analysis target

The monitoring data acquired during the excavation of a foundation pit mainly include the deep horizontal displacement of the soil (by inclinometer), vertical displacement of the retaining structure, ground surface settlement, groundwater level, etc. Considering that the current project is rich in groundwater and that the excavation area of the foundation pit comprises mainly a soft soil backfilling area, many factors (the vehicle load, excavation, accumulation load, etc.) can affect the settlement of the ground surface, so the amount of surface settlement should not be used as the control parameter to guide the excavation of the foundation pit. In contrast, the vertical displacement of the piles is affected mainly by the bottom heave of the pit, which cannot fully reflect the safety of the excavation process; therefore, the vertical pile displacement is similarly not listed as the target of back analysis. The monitored value of the deep horizontal displacement of the soil is affected only slightly by the temperature and other factors but is closely related to the stability of the foundation pit; hence, the deep horizontal displacement is suitable for the inversion target of this project. At present, the traditional displacement inversion method often takes the total horizontal displacement of the soil over the whole excavation depth as the inversion target and always converges to the local optimal solution of the objective function^27^, which means that the error between the small displacement and the value predicted by inversion is small but that the error between the maximum displacement and the value predicted by inversion is large. In fact, the maximum value and depth of deep horizontal displacement have the greatest impacts on construction, as these two parameters can directly reflect the safety of the foundation pit. Hence, the maximum value and the depth of the deep horizontal displacement of the soil are taken as the inversion target .

### Back analysis parameter

The foundation pit excavation is deep, and there are many excavation steps involving multiple layers of soil at the site. The physical and mechanical parameters of each soil layer are quite different, which increases the number of parameters to be inverted. Considering this kind of multivariable problem with multiple parameters and multiple excavation steps, transforming this problem into a single-variable problem for analysis and calculation can greatly improve the calculation efficiency. In the calculation process of single-variable optimization analysis, only one variable needs to be changed.

**Figure 6:**
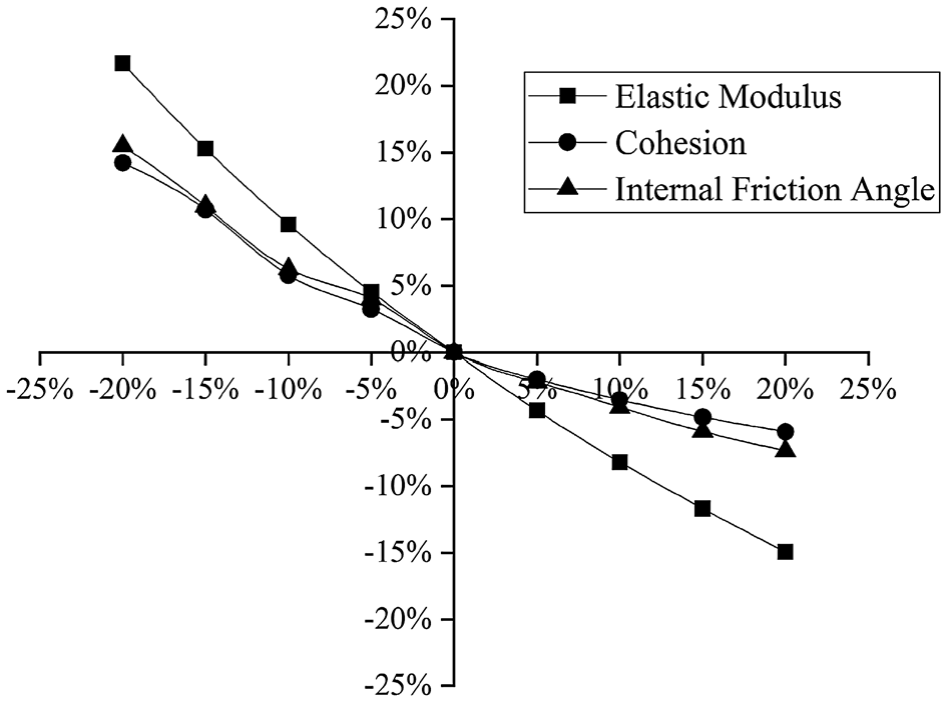
Relationships between the soil parameters and the horizontal displacement of the pile top.

**Figure 7:**
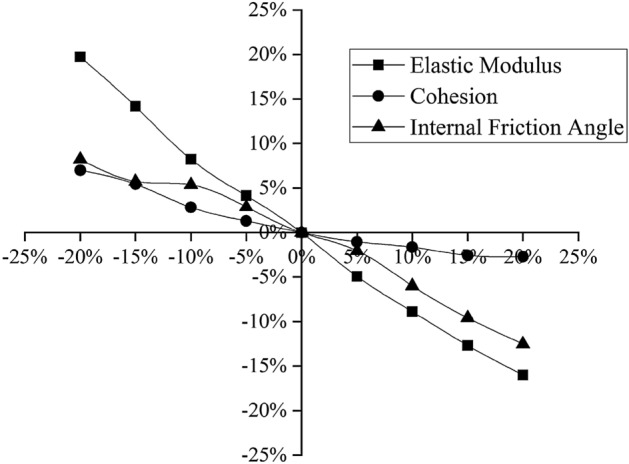
Relationships between the soil parameters and the vertical displacement of the pile.

**Figure 8:**
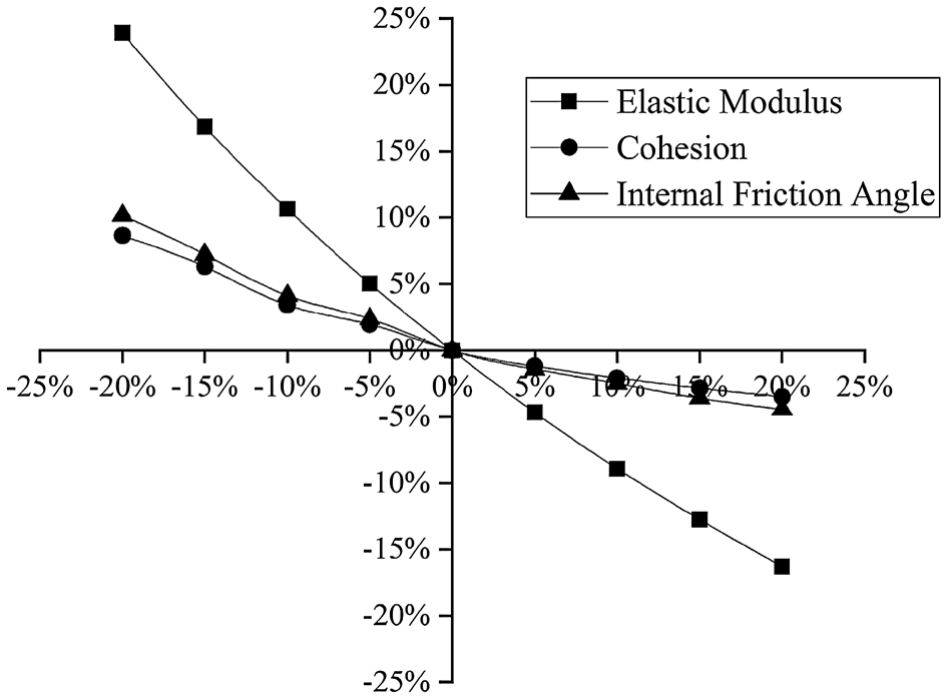
Relationships between the soil parameters and the maximum deep horizontal displacement of the soil layer.

The soil layer parameters involved in the model are mainly the elastic modulus *E*, the cohesion *c* and the internal friction angle *φ*. To simplify the calculation, taking section 4 as an example, this paper performs a sensitivity analysis^28,29^ to discuss the degree to which each parameter influences the excavation of the foundation pit, from which the most influential parameter is selected as the inversion parameter. The geological exploration report gives only the compressibility modulus *E*_*s*_; according to engineering experience in the Yangtze River Delta, the elastic modulus *E* of the soil is generally 2.5–3.5 times^30^ the compressibility modulus *E*_*s*_, and the initial elasticity-compressibility ratio of the project is *ξ*_0_ = 2.5; that is, *E* = 2.5*E*_*s*_. According to the geological exploration report, selecting the depth at which the maximum horizontal displacement of the deep layer, assuming that *E, c,* and $\varphi$ vary by the same amplitude from − 20% to 20%, their influences on the horizontal displacement of the pile top, the vertical displacement of the pile and the maximum deep horizontal displacement of the soil layer of the foundation pit are analyzed. The results are shown in Figs. 6, 7, 8.

As shown in the above three figures, the elastic modulus *E* has the greatest influence on the horizontal and vertical displacements of the pile top and the maximum deep horizontal displacement of the soil layer, and the degree of influence is more than 20%. Hence, the sensitivity of the elastic modulus *E* is considered to be larger than the sensitivities of the cohesion *c* and the internal friction angle *φ*. The elastic modulus *E* has the highest sensitivity and is thus taken as the main parameter for inversion analysis. The elastic modulus *E* of each soil layer is proportional to the compressibility modulus *E*_*s*_ in the geological exploration report, so the elasticity-compressibility ratio *ξ*_*i*_ (the ratio of *E* to *E*_*s*_) is taken as the specific inversion parameter.

## Foundation pit: inversion, prediction, reinversion and reprediction

According to the above analysis, the inversion parameter of this project is the elasticity-compressibility ratio, and the inversion target is the maximum deep horizontal displacement. For the elasticity-compressibility ratio, the following assumptions are made: the upper limit value *ξ*_*imax*_ is 10, the lower limit value *ξ*_*imin*_ is 1, and the initial value of *k* is 2.5. The number of soil layers to be inverted is determined according to the excavation range of each section. The inverted soil layers of each section are set to the next layer of soil where the excavation surface is located.

Inversion, prediction, re-inversion and re-prediction will be conducted based on the methods explained previously. The analysis sequence is consistent with the construction sequence, which means that the excavation of the foundation pit proceeds from shallow to deep (section 1–section 2–section 3–section 4). The inversion parameter, the actual elasticity-compressibility ratio of the soil layer, is used to predict the maximum deep horizontal displacement caused by the excavation of the foundation pit in the next section and to predict the possible risks and risk locations in advance to guide the safe construction of the foundation pit.

### Section 1

Because section 1 has only one excavation step, the excavation step weighting *h*_*ij*_ is not considered. The excavation depth is 4.27 m, and the excavation surface is in the second layer of soil, so the excavation influence area is set to the third layer, and the elasticity-compressibility ratio of the first three soil layers *ξ*_1_, *ξ*_2_ and *ξ*_3_ are taken as the inversion parameters.

Through the inversion calculation based on the field monitoring data, the interval influence coefficients *e*_*i*1_, the soil layer weightings *g*_*i*1_, and the final optimized values of the elasticity-compressibility ratio of the first three soil layers *ξ*_*i*_ of section 1 are shown in Table 5.

**Table 5:** Section 1 calculation parameters.

Soil layers	*e*_*i*1_	*g*_*i*1_	*ξ*_*i*_
1	0.047	0.288	3.4
2	0.073	0.444	5.3
3	0.044	0.268	3.2

**Figure 9:**
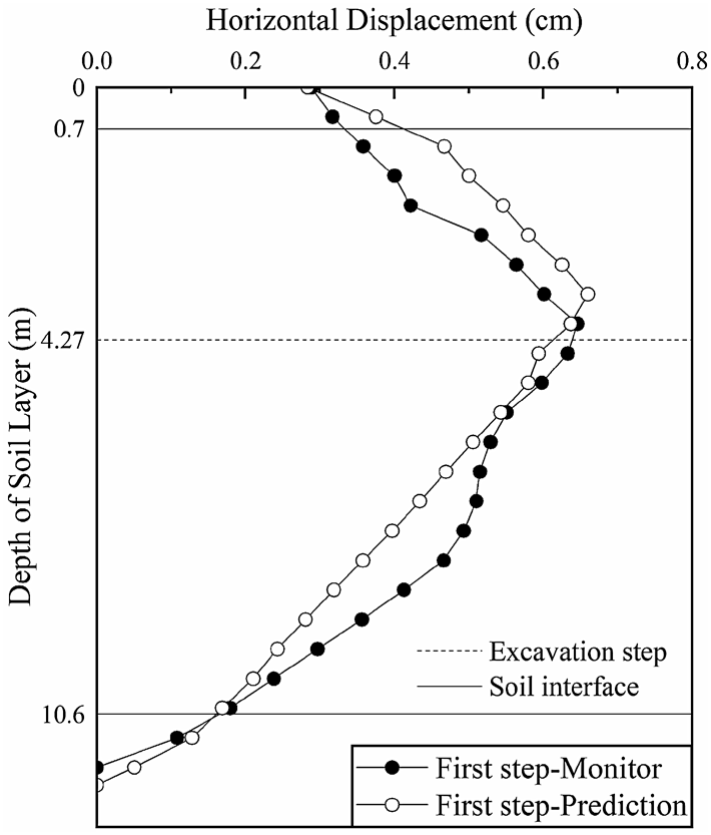
Deep horizontal displacement of section 1 after the inversion.

A comparison between the model simulation results and monitored data of section 1 after the parameter inversion is shown in Fig. 9 (the black solid lines in the figure represent the interface between two adjacent soil layers and the dotted line represents the location of excavation step).

After the inversion, the predicted change trend and magnitudes of the deep horizontal displacements of section 1 are consistent with those of the monitored values. The maximum deep horizontal displacement is 0.66 cm (occurring at a depth of 3.5 m), which is consistent with the maximum monitored value of 0.646 cm (occurring at a depth of 4 m). Hence, the soil layer elasticity-compressibility ratio inverted based on section 1 is taken as the prediction parameter of the next excavation section to analyze the variation in the deep displacement of the soil during the excavation of the foundation pit.

### Section 2

Section 2 is excavated in two steps, with excavation depths of 4.46 m and 6.67 m. The excavation surfaces of the two excavation steps are in the second layer of soil. Considering that the excavation surface of the second step is close to the bottom of the second layer of soil, the excavation influence area in the calculation model is set to the fourth layer.

**Figure 10:**
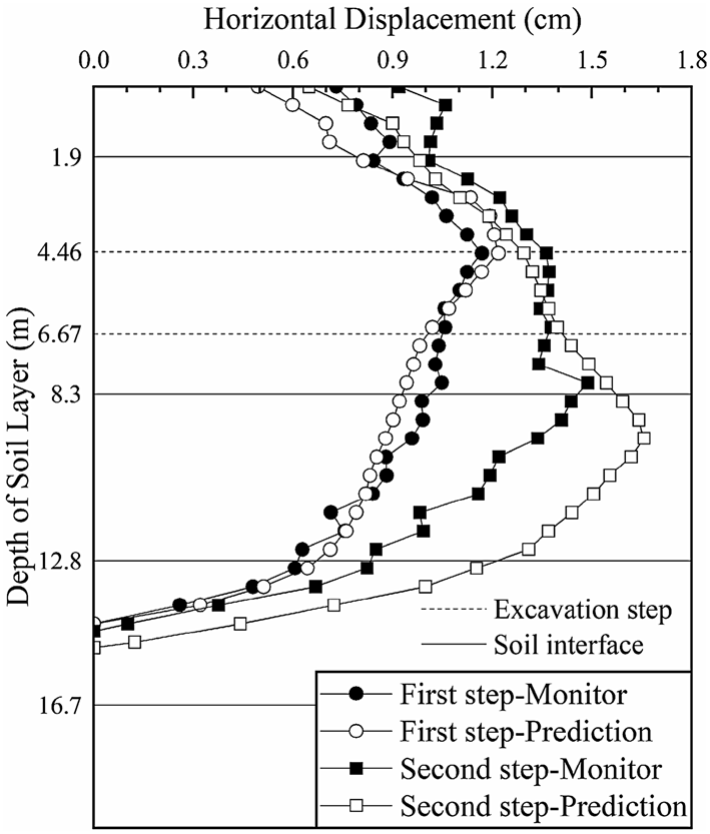
Deep horizontal displacement of section 2 before the inversion.

The elasticity-compressibility ratios of the first three layers obtained from the section 1 inversion are input into the numerical simulation calculation model of section 2. The deep horizontal displacement profile of the soil layers in section 2 is shown in Fig. 10 (the black solid lines in the figure represent the interface between two adjacent soil layers and the dotted lines represent the location of the excavation steps).

After the first excavation step, the predicted displacements of the soil are relatively consistent with the actual monitored values, confirming that the inversion calculation of section 1 is reasonable and effective and that *ξ*_1_, *ξ*_2_ and *ξ*_3_ obtained in the inversion calculation of section 1 are taken as the initial input for the calculation of section 2. After the second excavation step, the prediction is better above a depth of 8 m, but worse at depths greater than 8 m. This is because section 1 does not invert the elasticity-compressibility ratio of the fourth layer of soil; instead, the ratio is assumed to be 2.5. Hence, the predicted displacement near the fourth layer has a large error relative to the actual value. We continue the inversion of the fourth layer *ξ*_4_ by integrating the monitoring data. Again, since only one parameter is inverted, the soil layer weighting *g*_*ij*_ is not considered.

As obtained by the inversion, the interval influence coefficients *e*_4*j*_, the excavation step weightings *h*_4*j*_, and the final optimized value of the elasticity-compressibility ratio of the fourth layer of soil *ξ*_4_ of section 2 are shown in Table 6.

**Table 6:** Section 2 calculation parameters.

Steps	*e*_4*j*_	*h*_4*j*_	*ξ*_4_
1	0.049	0.331	5.34
2	0.101	0.669	

**Figure 11:**
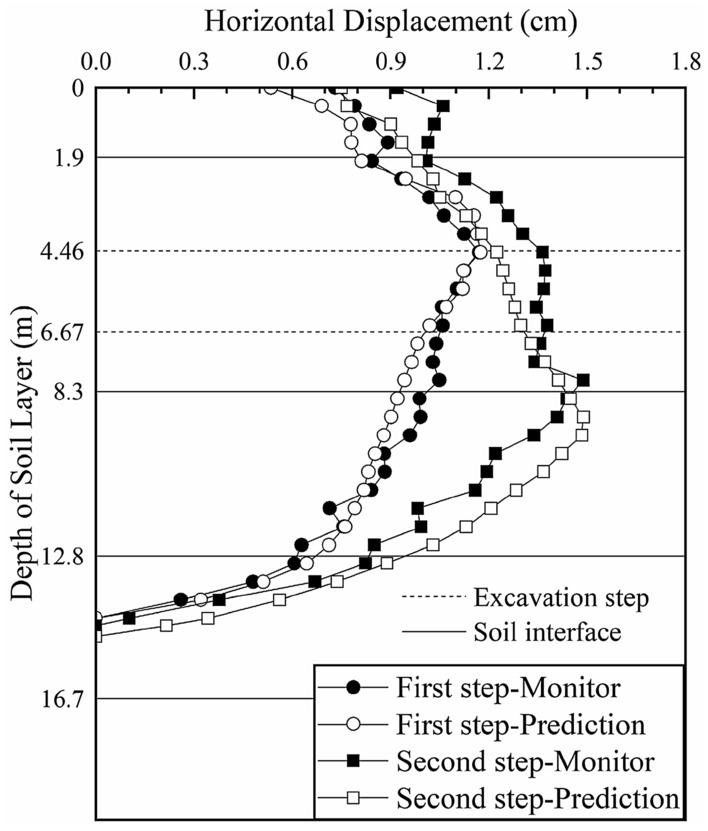
Deep horizontal displacement of section 2 after the inversion.

A comparison between the model simulation results and monitoring data of section 2 after the parameter inversion is shown in Fig. 11 (the black solid lines in the figure represent the interface between two adjacent soil layers, and the dotted lines represent the locations of the excavation steps).

After further inversion, the predicted deep horizontal displacements of section 2 are more consistent with the monitored values. After the first excavation step, the maximum deep horizontal displacement of the soil layer is 1.173 cm (occurring at a depth of 4.5 m), which is consistent with the maximum monitored value of 1.169 cm (occurring at a depth of 4.5 m); the difference is only 0.04 cm, which is reduced by 7.8% compared with the difference of 0.051 cm before inversion. After the second excavation step, the maximum deep horizontal displacement is 1.489 cm (occurring at a depth of 9 m), which is consistent with the monitored value of 1.488 cm (occurring at a depth of 8 m). The difference was only 0.001 cm, which is reduced by 99.4% compared with the difference of 0.618 cm before inversion. The elasticity-compressibility ratios of the first four soil layers are taken as the prediction parameters of the next excavation section to analyze the change trend of the deep displacement of the soil during the excavation of the foundation pit and to guide the safe construction of the foundation pit.

### Section 3

Section 3 is excavated in three steps, with excavation depths of 4.46 m, 7.26 m and 10 m. The excavation surfaces of the first and second excavation steps are in the second layer of soil, while the excavation surface of the third excavation step is in the fourth layer. Hence, the excavation influence area in the calculation model is taken as the fifth layer.

**Figure 12:**
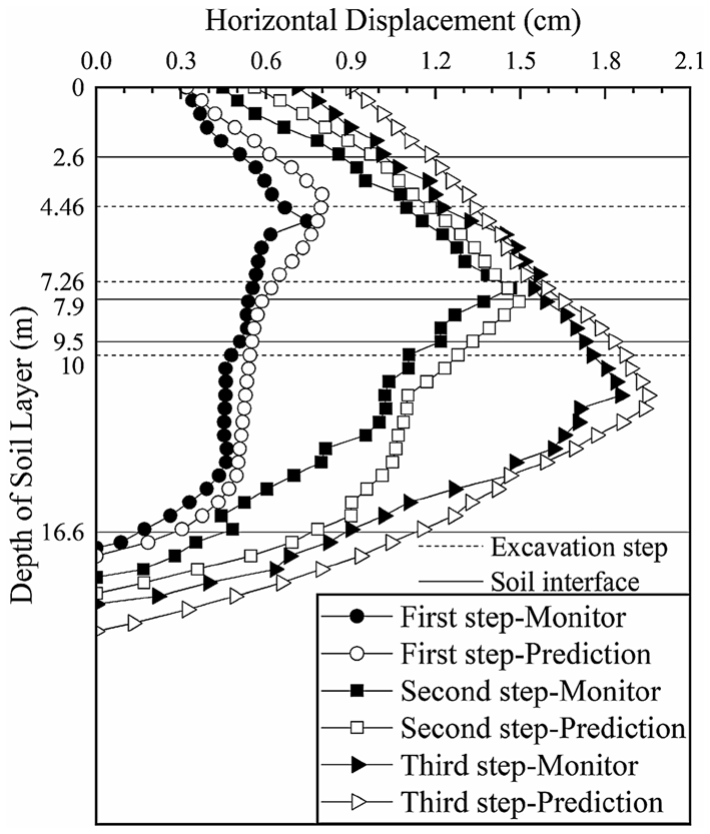
Deep horizontal displacement of section 3 before the inversion.

We input the soil parameters obtained from the previous two inversions into the calculation model of section 3 and predict the deep horizontal displacements of the soil layer, as shown in Fig. 12 (the black solid lines in the figure represent the interface between two adjacent soil layers and the dotted lines represent the locations of the excavation steps).

The predicted change trend and magnitudes of the displacements of the first four soil layers are consistent with those of the actual monitored values, which shows that the inversion results for sections 1 and 2 are reasonable and effective and that *ξ*_1_, *ξ*_2_, *ξ*_3_ and *ξ*_4_ obtained in the inversion calculation of section 1 are taken as the initial input for the calculation of section 3. However, with increasing depth (up to 14 m), the error between the predicted displacement and the actual monitored value increases. This error occurs predominantly in the fifth soil layer. To guide the subsequent construction, we continue the inversion of the fifth soil layer *ξ*_5_ by integrating the monitoring data. Only one parameter is inverted, so the soil layer weighting *g*_*ij*_ is not considered.

As obtained by the inversion, the interval influence coefficients *e*_5*j*_, the excavation step weightings *h*_5*j*_, and the final optimized value of the elasticity-compressibility ratio of the fifth layer of soil *ξ*_5_ of section 3 are shown in Table 7.

**Table 7:** Section 3 calculation parameters.

Steps	*e*_5*j*_	*h*_5*j*_	*ξ*_5_
1	0.017	0.081	2.58
2	0.066	0.314	
3	0.127	0.605	

**Figure 13:**
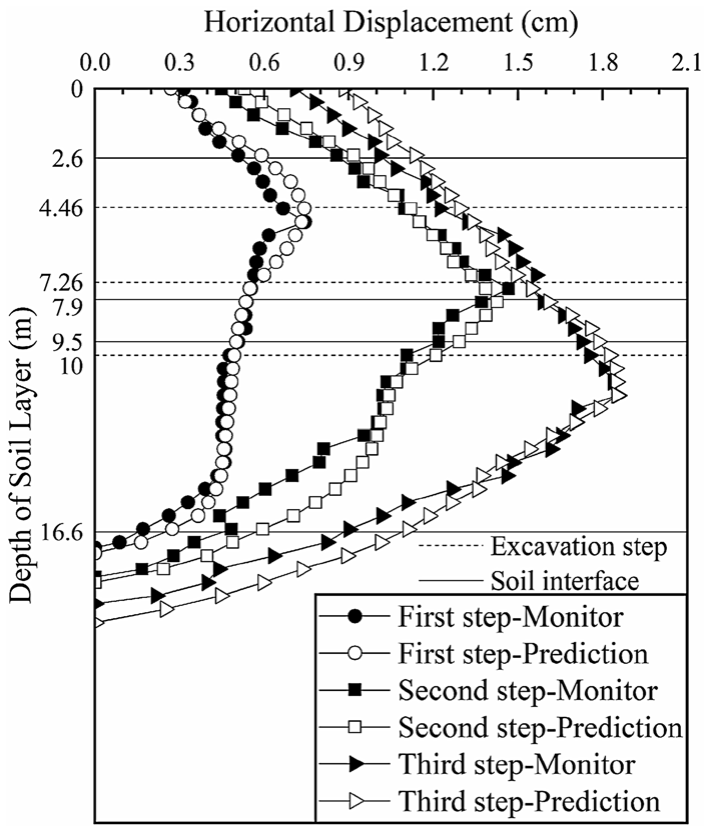
Deep horizontal displacement of section 3 after the inversion.

A comparison between the model simulation results and monitoring data of section 3 after the parameter inversion is shown in Fig. 13 (the black solid lines in the figure represent the interface between two adjacent soil layers and the dotted lines represent the locations of the excavation steps).

After further inversion, the predicted change trend and magnitudes of the deep horizontal displacement of section 3 are more consistent with those of the monitored values. After the first excavation step, the maximum deep horizontal displacement of the soil layer is 0.742 cm (occurring at a depth of 4.5 m), which is consistent with the maximum monitored value of 0.745 cm (occurring at a depth of 5 m). The difference was 0.003, which is reduced by 95% compared with the difference of 0.053 cm before inversion. After the second excavation step, the maximum deep horizontal displacement is 1.425 cm (occurring at a depth of 8 m), which is slightly smaller than the monitored value of 1.466 cm (occurring at a depth of 7.5 m) but essentially consistent. After the third excavation step, the maximum deep horizontal displacement is 1.851 cm (occurring at a depth of 11.5 m), which is consistent with the maximum monitored value of 1.854 cm (occurring at a depth of 11.5 m). The difference is 0.003, which is reduced by 97% compared with the difference of 0.097 cm before inversion. The elasticity-compressibility ratios of the first five soil layers are taken as the prediction parameters of the fourth excavation section to analyze the change trend of the deep displacement of the soil during the excavation of the foundation pit and to guide the safe construction of the foundation pit.

### Section 4

Section 4 is excavated in three steps, with excavation depths of 6.5 m, 11 m and 14 m. The excavation surface of the first excavation is in the second layer of soil, the excavation surface of the second excavation is in the third layer, and the excavation surface of the third excavation is at the bottom of the fourth layer. Because the depth of each excavation of section 4 is large, increasing the risks associated with the foundation pit, the excavation influence area is taken to be the sixth layer of soil.

**Figure 14:**
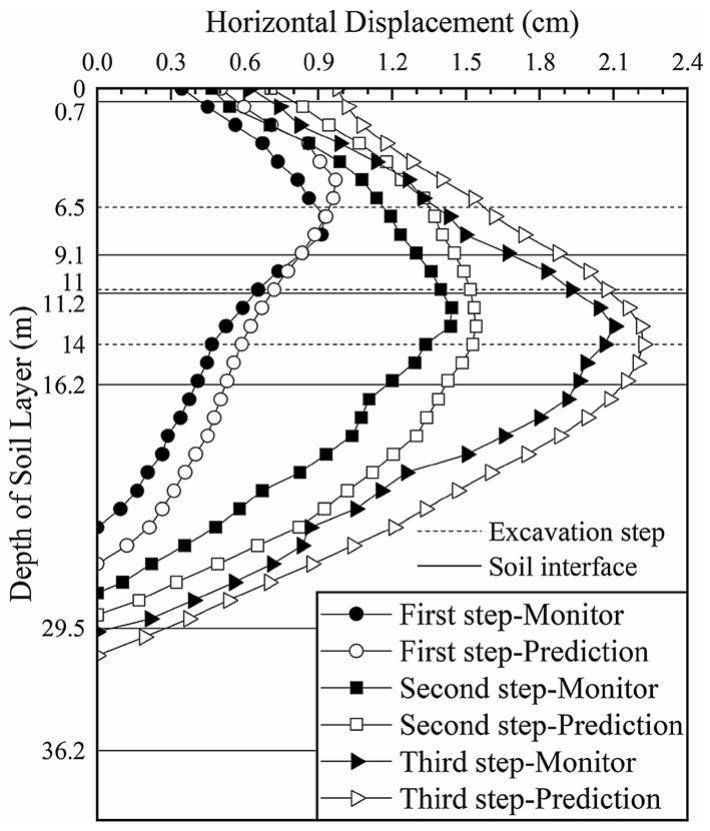
Deep horizontal displacement of section 4 before the inversion.

We input the soil parameters obtained from the previous three inversions into the calculation model of section 4 and predict the deep horizontal displacement of the soil layer, as shown in Fig. 14 (the black solid lines in the figure represent the interface between two adjacent soil layers and the dotted lines represent the locations of the excavation steps).

The predicted change trend and magnitudes of the displacements of the first four layers and the upper half of the fifth layer are consistent with those of the actual monitored values, verifying that the inversion results of sections 1, 2 and 3 are reasonable and effective. The lower half of the fifth layer of soil is affected by the sixth layer of soil without inversion, which results in an increase in the error between the predicted and monitored values of the deep horizontal displacement of the soil. Combined with the monitoring data, we continue to invert the elasticity-compressibility ratio of the sixth layer of soil. Only one parameter is inverted, so the soil layer weighting *g*_*ij*_ is not considered.

As obtained by inversion calculation, the interval influence coefficients *e*_6*j*_, excavation step weightings *h*_6*j*_, and final optimized value of the elasticity-compressibility ratio of the sixth layer *ξ*_6_ of section 4 are shown in Table 8.

**Table 8:** Section 4 calculation parameters.

Steps	*e*_6*j*_	*h*_6*j*_	*ξ*_6_
1	0.195	0.416	3.09
2	0.145	0.309	
3	0.129	0.275	

**Figure 15:**
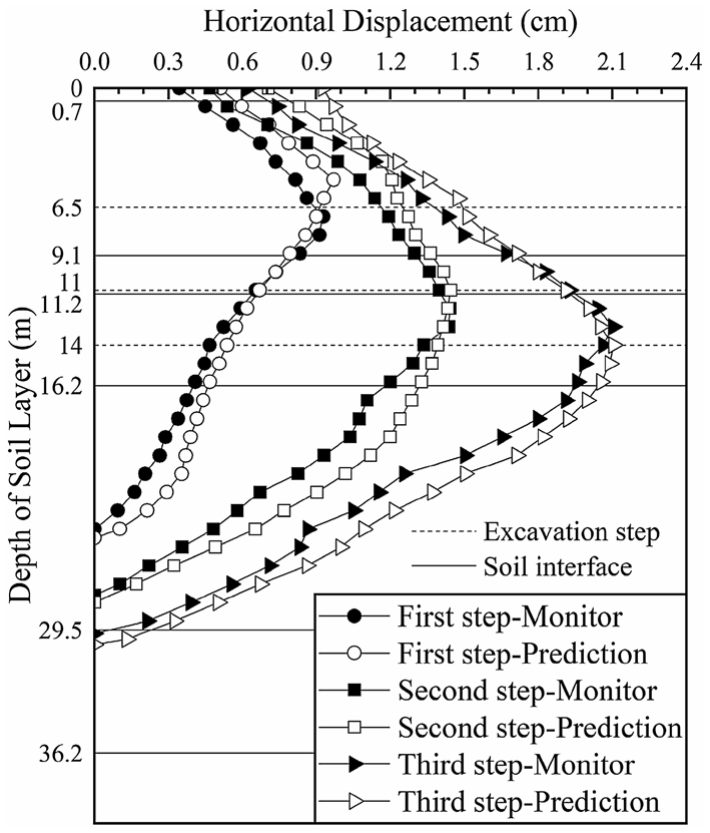
Deep horizontal displacement of section 4 after the inversion.

A comparison between the model simulation results and monitoring data of section 4 after the parameter inversion is shown in Fig. 15 (the black solid lines in the figure represent the interface between two adjacent soil layers and the dotted lines represent the locations of the excavation steps).

After further inversion, the predicted change trend and magnitudes of the deep horizontal displacement of section 4 was more consistent with those of the monitored value. After the first excavation step, the maximum deep horizontal displacement of the soil layer is 0.940 cm (occurring at a depth of 5.5 m), which is slightly larger than the monitored value of 0.927 cm (occurring at a depth of 7 m) but essentially consistent. The difference was 0.013, which is reduced by 68.9% compared with the difference of 0.097 cm before inversion. After the second excavation step, the maximum deep horizontal displacement is 1.445 cm (occurring at a depth of 11 m), which is consistent with the maximum monitored value of 1.441 cm (occurring at a depth of 12 m). The difference was 0.004, which is 95.9% less than the difference of 0.099 before inversion. After the third excavation step, the maximum deep horizontal displacement is 2.106 cm (occurring at a depth of 14 m), which is consistent with the maximum monitored value of 2.104 cm (occurring at a depth of 13 m). The difference was 0.002, which is 98.2% less than the difference of 0.116 before inversion.

## Discussion

In this paper, only sections with excavation depths shallower than 14 m are analyzed, whereas sections with deeper excavation depths are not studied. This is because the buried depth of the inclinometer pipe is insufficient in regions with a large excavation depth, and the deep horizontal displacement value of the soil cannot be monitored due to the movement of the bottom of the inclinometer pipe. Hence, the rationality of the predicted results cannot be verified through a comparison with the actual displacements. However, through the inversion and prediction of the four sections, the proposed method is found to be suitable to predict the deformation of cantilevered deep foundation pits during the excavation process.

Note that the established plane strain model cannot readily analyze the interaction between adjacent construction sections, and the inversion calculation efficiency is not sufficiently high. Hence, one of the future research directions is to establish a 3D dynamic inversion analysis method in conjunction with an intelligent algorithm.

In addition, the depth of foundation pit excavation involves multiple soil layers; thus, the physical and mechanical parameters vary greatly, which increases the difficulty of inversion. Hence, this paper considers only the soil parameters and deformation parameters relevant to the project and analyzes a single inversion target and a single inversion parameter. Optimization analysis methods involving multiple targets and multiple parameters are future research directions. Concurrently, the difference between the results of the numerical analysis and the actual situation caused by the adopted bracings, soil-structure interface modeling technique and the soil constitutive law are another research direction to explore in the future.

## Conclusion

By establishing a numerical simulation model of foundation pit excavation, an analysis method comprising inversion, prediction, reinversion and reprediction is proposed to predict the maximum value and the depth of the deep horizontal displacement of a soil layer caused by foundation pit excavation based on monitoring data. The following main points can be obtained in conclusion:

1. According to the relationship between soil parameters and the maximum horizontal displacement of the enclosure pile, the elastic modulus *E* has the greatest influence on the horizontal displacement of the enclosure pile top and the maximum horizontal displacement of the vertical displacement of the pile top. Therefore, the elastic modulus *E* is considered to be more sensitive and is used as the main parameter for inversion analysis.
2. Through the inversion of excavated soil parameters in different soil layers, the accurate values of the prediction results are significantly higher than those before the inversion. According to the final monitoring and prediction results, the least squares method is used as the overall error evaluation index. After the inversion, the overall error of section 2 decreases from 1.52 (before inversion) to 0.498 (after inversion), decreasing by 67.24%. The overall error of section 3 decreases from 0.79 (before inversion) to 0.47 (after inversion), decreasing by 40.5%. The overall error of section 4 decreases from 1.51 (before inversion) to 098 (after inversion), decreasing by 35%. The prediction curves of the deep horizontal displacement of the soil are all close to the monitoring displacement curves, which play a good guiding role in foundation pit excavation and ensure the safe construction of the foundation pit.

## Data Availability

The data used to support the findings of this study are available from the corresponding author upon request.
